# COVID-19 monoclonal antibody treatment impact on symptoms and post-COVID conditions among high-risk patients at a Federally Qualified Health Center

**DOI:** 10.1186/s12879-023-08057-7

**Published:** 2023-02-22

**Authors:** Natalie L. Vawter, Job G. Godino, Sydney V. Lewis, Adam W. Northrup, Jane C. Samaniego, Jacqueline Y. Poblete, Jesus A. Guereca, Sydney P. Sharp, Eva Matthews, Noe C. Crespo, Pauline G. Lucatero, Monica M. Vidaurrazaga, Christian B. Ramers

**Affiliations:** 1 Laura Rodriguez Research Institute, Family Health Centers of San Diego, 1750 5th Ave, San Diego, CA 92101 USA; 2 Herbert Wertheim School of Public Health and Human Longevity Science, University of California San Diego, San Diego, CA USA; 3 School of Public Health, San Diego State University, San Diego, CA USA; 4 Family Health Centers of San Diego, San Diego, CA USA

**Keywords:** COVID-19, Monoclonal antibodies, COVID-19 symptoms, Post-COVID conditions

## Abstract

**Background:**

Monoclonal antibody (mAb) treatment for COVID-19 is associated with improved clinical outcomes. However, there is limited information regarding the impact of treatment on symptoms and the prevalence of post-COVID Conditions (PCC). Understanding of the association between time to mAb infusion and the development of PCC is also limited.

**Methods:**

This longitudinal study was conducted among patients with COVID-19 who received mAb infusions at a Federally Qualified Health Center in San Diego, CA. A series of telephone interviews were conducted at baseline and follow-up (14 days and 28+ days). A comprehensive symptom inventory was completed and physical and mental health status were measured using PROMIS-29 and PHQ-2. Pearson’s Chi-squared tests and independent two-sample t-tests were performed to test for association between time to mAb infusion and outcomes at follow-up. A Poisson regression model was used to analyze whether time to mAb infusion predicts risk of developing PCC.

**Results:**

Participants (N = 411) were 53% female, ranged in age from 16 to 92 years (mean 50), and a majority (56%) were Latino/Hispanic. Cross-sectional findings revealed a high symptom burden at baseline (70% of patients had cough, 50% had fever, and 44% had headache). The prevalence of many symptoms decreased substantially by the final follow-up survey (29% of patients had cough, 3% had fever, and 28% had headache). Longitudinal findings indicated that 10 symptoms decreased in prevalence from baseline to final follow-up, 2 remained the same, and 14 increased. The severity of symptoms and most patient-reported physical and mental health measure scores decreased over time. The prevalence of PCC was 69% when PCC was defined as ≥ 1 symptom at final follow-up. Time to mAb infusion was not significantly associated with any outcome at follow-up. Time to infusion was not associated with PCC status at final follow-up in the crude or adjusted Poisson regression models.

**Conclusions:**

The prevalence of PCC was high among this patient population following COVID-19 mAb treatment. Time to mAb infusion did not predict the development of PCC. Further research in these areas is essential to answer urgent clinical questions about effective treatments of COVID-19.

## Introduction

As of September 2022, there have been over 94 million coronavirus disease 2019 (COVID-19) cases in the United States (US), resulting in over one million deaths [1] In response to the surging burden of morbidity and mortality, the US Food and Drug Administration (FDA) issued emergency use authorizations (EUAs) for monoclonal antibodies (mAbs) for treatment of COVID-19, beginning with Bamlanivimab, Casirivimab, and Imdevimab [2, 3]. Through binding to the spike protein of the severe acute respiratory syndrome coronavirus 2 (SARS-CoV-2) virus, mAbs support the body’s natural immune response and prevent further invasion of the virus [4]. Monoclonal antibody (mAb) infusions have been associated with a reduced prevalence of emergency room visits and hospitalizations in the weeks immediately following onset of COVID-19 [5–9]. Shorter time from symptom onset to mAb infusion has also been associated with a reduced likelihood of hospitalization in patient populations of solid organ transplant recipients and high-risk outpatients (defined by the following criteria: age ≥ 65; body mass index [BMI] ≥ 35 kg/m^2^; diabetes; chronic kidney disease [CKD]; immunosuppressant disease or treatment; age ≥ 55 with hypertension, chronic respiratory disease/chronic obstructive pulmonary disease, or cardiovascular disease [CVD]; or age 12–17 and meeting specific criteria) [10, 11]. This suggests that time to infusion (TTI) may be important to improving clinical outcomes.

While mAbs have shown encouraging results improving health-related outcomes immediately following infection, many patients experience new, recurring, or persistent symptoms of COVID-19 for weeks or even months after disease onset. Currently, the Centers for Disease Control and Prevention (CDC) refers to this phenomenon as post-COVID Conditions (PCC), “an umbrella term for the wide range of health consequences that are present four or more weeks after infection with SARS-CoV-2” [12]. The World Health Organization (WHO) defines PCC as occurring “in individuals with a history of probable or confirmed SARS CoV-2 infection, usually 3 months from the onset of COVID-19 with symptoms that last for at least 2 months and cannot be explained by an alternative diagnosis” [13]. Scientific understanding of the diagnoses, phenotypes, and epidemiology of PCC is evolving and the burden associated with PCC is increasing—estimates of PCC prevalence among previously infected adults range from 4.7 to 80% [14]. Although the experience of PCC is heterogeneous across patients, commonly reported symptoms include fatigue, headaches, shortness of breath, chest pain, and loss of smell [14–17]. In many patients, the reported persistent symptoms (1) are highly debilitating, (2) result from general complications of illness and hospitalization, (3) are secondary to organ system damage, and/or 4) are of unclear etiopathogenesis [18].

In research exploring factors associated with the development of PCC, preliminary evidence suggests that PCC is more common among patients of female sex and older age, though some studies note that the significance of advanced age decreases when adjusting for comorbidities and severity of illness [14–16, 19, 20]. The development of PCC has also been associated with a higher number of symptoms at illness onset, a higher severity of initial symptoms, and hospitalization [14–16, 20, 21]. As research focus on mAb infusion treatment and PCC intensifies, there remains a critical need to advance understanding of the changes in symptoms and prevalence of PCC among patients who have undergone mAb infusion treatment. Furthermore, there is a need for examining the role that time to mAb infusion may play in the development of PCC. To these ends, the present study explored (1) how symptoms and patient-reported physical and mental health measures change over time following mAb treatment, (2) whether time to mAb infusion is associated with patient outcomes at follow-up, (3) the prevalence of PCC in mAb-treated patients, and (4) whether time to mAb infusion predicts risk of developing PCC. We hypothesized that a shorter time to mAb infusion would be associated with improved patient outcomes and reduce the risk of PCC.

## Methods

### Study population and design

This study was an observational, longitudinal study to assess patients’ symptoms and physical and mental health measures following mAb infusion. Patients with COVID-19 who received mAb therapy at the mAb infusion clinic at Family Health Centers of San Diego (FHCSD) were eligible to participate in the study. FHCSD is a federally qualified health center (FQHC) located in San Diego, California, US. The mAb infusion clinic at FHCSD provides outpatient COVID-19 treatment with the goal of reducing the risk of severe disease, hospitalization, and death. According to the EUA of mAb agents during the study period, patients were eligible to receive mAb infusion up to 10 days following COVID-19 symptom onset. Providers making treatment decisions used the Monoclonal Antibody Screening Score (MASS) and National Institutes of Health (NIH) guidelines to prioritize mAb treatment [22, 23]. The MASS score was developed at Mayo Clinic in Minnesota and incorporates age, comorbidities, and immunocompromised status [22]. Providers essentially only treated patients with MASS > 1 and in the busiest times with scarcity of resources, patients were prioritized by MASS (i.e., patients with higher MASS would receive appointments before those with lower MASS) and NIH tier, incorporating the NIH COVID Treatment Guidelines table on Prioritization of Therapies when there were logistical and supply constraints [23]. This scheme, which categorizes patients in tiers based on immunocompromised status, vaccination status, age, and comorbidities, was also useful, since patients that didn't fit into Tier 1–4 would also be those with MASS Score of 0, and generally would not receive treatment. The nature of the public health crisis necessitated that providers make therapeutic decisions based on their best clinical judgement.

Six employees at the Laura Rodriguez Research Institute (LRRI) at FHCSD conducted a series of telephone interviews among eligible patients at baseline and at follow-up (14 days and 28+ days). All interviewers followed a standard operating procedure and interview script to ensure consistency. Surveys were conducted in English or Spanish. All patients (or parents/legal guardians of patients < 18 years) included in the study completed FHCSD’s broad informed consent form which includes a specific authorization for the use of de-identified health information for population health and quality improvement studies. The study was reviewed and approved by the Institutional Review Board at San Diego State University (HS-2022-0113).

The COVID mAb Baseline Survey collected the date of symptom onset and date of mAb infusion and measured the presence of 30 symptoms at the time the patient was first seen as a dichotomous variable (“no” or “yes”). Two follow-up surveys were conducted: the COVID mAb Follow-up Day 14 Survey (D14) and the COVID mAb Follow-up Day 28+ Survey (D28+). The D14 and D28+ surveys were identical and measured the presence and severity of 26 symptoms at follow-up as categorical variables (e.g., “none,” “mild,” “moderate,” or “severe”). The D14 and D28+ surveys also included the Patient Health Questionnaire-2 (PHQ-2) and the Patient-Reported Outcomes Measurement Information System-29 (PROMIS-29) profile (v2.0).

### Measures

#### Symptoms

Symptom outcomes included the presence of symptoms at baseline and follow-up (measured by all three surveys) as well as the severity of symptoms at follow-up (measured by D14 and D28+).

#### Patient-reported physical and mental health measures

Patient-reported physical and mental health outcomes included PHQ-2 Total Score and PROMIS-29 Domain Scores at follow-up. The validity of PHQ-2 has been demonstrated [24]. The PROMIS-29 profile v2.0 assesses seven physical and mental health domains including Physical Function, Anxiety, Depression, Fatigue, Sleep Disturbance, Social Roles, and Pain Interference [25]. PROMIS measures have demonstrated validity across a range of clinical populations and have been used to assess the trajectory of COVID-19 [26–28].

#### Time to mAb infusion

The potential predictor of interest in multiple analyses was TTI, defined as the time from COVID-19 symptom onset to mAb infusion. This variable was created using the date of symptom onset and date of mAb infusion (both collected in the Baseline Survey). Time to infusion was defined as a binary variable for the purpose of analysis—a cutpoint of 6 days was chosen prior to the analyses based on the distribution of the TTI variable. Analyses compared two groups, patients who received mAb infusion 1 to 6 days following COVID-19 symptom onset and patients who received mAb infusion 7 to 10 days following symptom onset.

#### Post-COVID conditions (PCC)

The prevalence of PCC was calculated, with PCC status at D28+ defined by six different definitions. Three definitions were based on the total number of symptoms at D28+: PCC Definition 1 (≥ 1 symptom), PCC Definition 2 (≥ 3 symptoms), and PCC Definition 3 (≥ 5 symptoms). Three definitions were based on the severity of symptoms at D28+: PCC Definition 4 (≥ 1 moderate or severe symptom), PCC Definition 5 (≥ 2 moderate or severe symptoms), and PCC Definition 6 (≥ 3 moderate or severe symptoms). These definitions were chosen prior to the analyses based on the distribution of the PCC variable.

#### Covariates

Additional variables of interest included selected demographic characteristics (sex assigned at birth, race, ethnicity, and age) and clinical characteristics (vaccination status, mAb infusion type, and number of symptoms at Baseline Survey). Age was defined as a binary variable for the purpose of analysis—a cutpoint of 50 years (≤ 50 years vs > 50 years) was chosen prior to the analyses. Number of symptoms at Baseline Survey was also defined as a binary variable—a cutpoint of 4 was chosen prior to the analyses.

### Statistical analysis

All analyses were performed in R (version 4.1.2) [47]. The following packages were used: data.table, dplyr, ggplot2, lmtest, msm, readxl, sandwich, and tidyverse [48–56]. Descriptive statistics (percentages, means, standard deviations, medians, and ranges) were used to describe the demographic characteristics of the study population overall as well as among patients who completed both D14 and D28+ and those who completed D28+ only. The prevalence of symptoms was calculated at baseline and follow-up and the severity of symptoms was characterized at follow-up. For patient-reported physical and mental health outcomes at follow-up, means and standard deviations were calculated according to PROMIS-29 Domain Scores and PHQ-2 Total Scores.

Pearson’s Chi-squared tests were performed to test for association between TTI (1–6 days vs 7–10 days) and categorical variables at D28+ (symptom count category, symptom severity category, and six definitions of PCC status). Symptom count category was defined by total number of symptoms at D28+ (0, 1–2, 3–4, or 5–20); symptom severity category was defined by total number of moderate or severe symptoms at D28+ (0, 1, 2, ≥ 3). For the PCC tests, Yates’ continuity correction was performed. The significance level (alpha) for Chi-squared tests was 0.05. Independent two-sample t-tests were performed to test for association between TTI and continuous outcomes at D28+ (PHQ-2 Total Score and PROMIS-29 Domain Scores). Means were compared between the two TTI groups and the equal variances assumption was tested. For outcomes that met the equal variances assumption, Student’s t-tests were performed; for outcomes that did not meet the equal variances assumption, Welch’s t-tests were performed. The Bonferroni correction was used to control for multiple testing, resulting in a significance level (alpha) of 0.00625 for the independent two-sample t-tests.

A Poisson regression model with robust error variances (i.e., “sandwich estimation”) was used to assess the hypothesized association between TTI and PCC status at D28+  [29, 30]. This method was selected because it enables direct estimation of prevalence ratios (i.e., relative risks) for a binary outcome. This measure of association was appropriate in this study and was preferable over odds ratios, since odds ratios approximate relative risk only for rare outcomes and positive PCC status was common in this study population [31]. The unadjusted (crude) model included TTI as the only predictor of PCC status. Model 1 included TTI and demographic variables (sex assigned at birth, race, ethnicity, and age). Model 2 included all variables in Model 1 plus clinical characteristics (vaccination status, mAb infusion type, and number of symptoms at Baseline Survey). The significance level (alpha) was set at 0.05 for all analyses unless otherwise noted.

## Results

From 12/31/2020 to 10/5/2021, 1447 high-risk patients with positive COVID-19 diagnosis received mAb infusion at FHCSD—these patients were eligible to participate in the survey study. Surveys were conducted from 6/8/2021 to 11/1/2021. Interviewers contacted 635 patients and 411 chose to participate. All 411 patients completed the Baseline Survey, 199 patients completed both D14 and D28+ , and 212 patients completed D28+ only. There was variation in follow-up times from symptom onset to D14 (mean 19 days; SD 3; median 19; range 13 to 29) and D28+ (mean 72 days; SD 52; median 43; range 21 to 186).

Demographic characteristics of the study population are shown in Table 1. The study population was 53% female and 47% male. Participants ranged in age from 16 to 92 (mean age: 50 years). In terms of race, 61% of participants were white and 10% were non-white. In terms of ethnicity, 56% of participants were Latino/Hispanic and 16% were non-Latino/Hispanic. Forty-four percent were FHCSD patients. Most participants lived in either Central (40%) or South (30%) San Diego. With respect to vaccination status pre-infusion, 34% of patients were fully vaccinated, 2% were partially vaccinated, and 64% were unvaccinated or had unknown vaccination status. The preferred language of most patients was English (52%) or Spanish (43%). The most prevalent chronic comorbidities included essential hypertension (47%), diabetes (30%), depressive disorders (20%), asthma (15%), and obesity (13%).

**Table 1:** Demographic characteristics among patients who completed both D14 and D28+ surveys vs only D28+ survey

	Day 14 and Day 28+ (N = 199)	Day 28+ only (N = 212)	Total (N = 411)
*Sex assigned at birth*			
Female	102 (51.3%)	117 (55.2%)	219 (53.3%)
Male	97 (48.7%)	95 (44.8%)	192 (46.7%)
*Age (years)*			
Mean (SD)	49.1 (15.9)	50.8 (15.5)	50.0 (15.7)
Median [Min, Max]	49.0 [16.0, 88.0]	51.0 [18.0, 92.0]	50.0 [16.0, 92.0]
*Age group*			
< 18	2 (1.0%)	0 (0%)	2 (0.5%)
18–29	20 (10.1%)	19 (9.0%)	39 (9.5%)
30–39	38 (19.1%)	30 (14.2%)	68 (16.5%)
40–49	41 (20.6%)	43 (20.3%)	84 (20.4%)
50–59	48 (24.1%)	62 (29.2%)	110 (26.8%)
60 +	50 (25.1%)	58 (27.4%)	108 (26.3%)
*Race*			
Non-White	15 (7.5%)	24 (11.3%)	39 (9.5%)
White	117 (58.8%)	132 (62.3%)	249 (60.6%)
Unknown	67 (33.7%)	56 (26.4%)	123 (29.9%)
*Ethnicity*			
Latino/Hispanic	93 (46.7%)	138 (65.1%)	231 (56.2%)
Non-Latino/Hispanic	36 (18.1%)	30 (14.2%)	66 (16.1%)
Unknown	70 (35.2%)	44 (20.8%)	114 (27.7%)
*San Diego Region*			
Central	75 (37.7%)	90 (42.5%)	165 (40.1%)
East	24 (12.1%)	33 (15.6%)	57 (13.9%)
North Central	28 (14.1%)	14 (6.6%)	42 (10.2%)
North Coastal	1 (0.5%)	2 (0.9%)	3 (0.7%)
North Inland	7 (3.5%)	4 (1.9%)	11 (2.7%)
Outside County	3 (1.5%)	1 (0.5%)	4 (1.0%)
South	59 (29.6%)	66 (31.1%)	125 (30.4%)
Unknown	2 (1.0%)	2 (0.9%)	4 (1.0%)
*FHCSD patient status*			
FHCSD	51 (25.6%)	128 (60.4%)	179 (43.6%)
Non-FHCSD	148 (74.4%)	84 (39.6%)	232 (56.4%)
*Primary care provider*			
FHCSD	51 (25.6%)	128 (60.4%)	179 (43.6%)
Kaiser Permanente	49 (24.6%)	17 (8.0%)	66 (16.1%)
Other	73 (36.7%)	41 (19.3%)	114 (27.7%)
San Ysidro Health	3 (1.5%)	3 (1.4%)	6 (1.5%)
Scripps	8 (4.0%)	12 (5.7%)	20 (4.9%)
Sharp	9 (4.5%)	10 (4.7%)	19 (4.6%)
UCSD	1 (0.5%)	1 (0.5%)	2 (0.5%)
VA	5 (2.5%)	0 (0%)	5 (1.2%)
*Vaccination status pre-infusion*			
Fully vaccinated	98 (49.2%)	43 (20.3%)	141 (34.3%)
Partially vaccinated	1 (0.5%)	8 (3.8%)	9 (2.2%)
Unvaccinated/unknown	100 (50.3%)	161 (75.9%)	261 (63.5%)
*Language*			
English	114 (57.3%)	98 (46.2%)	212 (51.6%)
Spanish	72 (36.2%)	103 (48.6%)	175 (42.6%)
Unknown	13 (6.5%)	8 (3.8%)	21 (5.1%)
Other	0 (0%)	3 (1.4%)	3 (0.7%)

	Day 14 and Day 28+ (N = 72)	Day 28+ only (N = 112)	Total (N = 184)
*Chronic comorbidities*^*a*^			
Essential hypertension	34 (47.2%)	52 (46.4%)	86 (46.7%)
Diabetes^b^	21 (29.2%)	34 (30.4%)	55 (29.9%)
Depressive disorders	9 (12.5%)	27 (24.1%)	36 (19.6%)
Asthma	9 (12.5%)	18 (16.1%)	27 (14.7%)
Obesity	11 (15.3%)	12 (10.7%)	23 (12.5%)

All patients also completed the Baseline Survey

*VA* Veterans Affairs, *SD* standard deviation, *FHCSD* Family Health Centers of San Diego, *UCSD* University of California, San Diego

^a^Chronic comorbidity data were mostly only available for internal patients, which were a minority of the study participants. Chronic comorbidities are not mutually exclusive

^b^Includes type 1 diabetes and type 2 diabetes

**Fig. 1:**
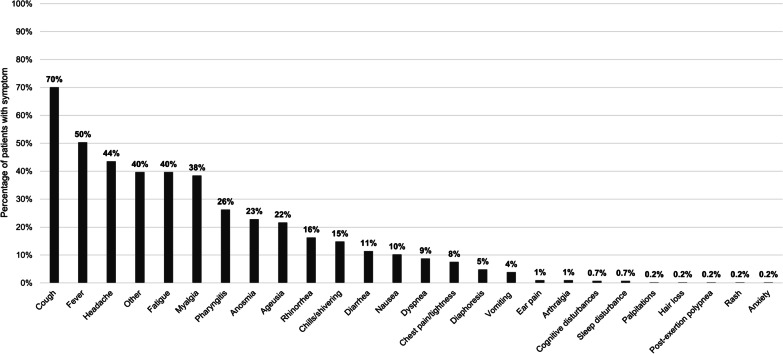
Prevalence of symptoms at baseline survey (N = 411). Data on the presence of the following symptoms were also collected, but no patients reported having them at baseline: thromboembolism, chronic kidney disease, need for oxygen, loss of hearing, and depression

At baseline, the five most prevalent symptoms were cough (70%), fever (50%), headache (44%), fatigue (40%), and “other” (40%) (Fig. 1). At D28+ , seven symptoms had a prevalence over 20%, including cough (29%), headache (28%), myalgia (26%), anosmia (24%), dyspnea (23%), post-exertion polypnea (21%), and ageusia (20%) (Table 2). At D28+ , the most common mild symptoms were cough (20%), anosmia (20%), ageusia (18%), headache (17%), and myalgia (15%). The most common moderate symptoms were headache (9%), myalgia (9%), cough (8%), dyspnea (8%), and post-exertion polypnea (7%). The most common severe symptoms were anosmia (3%), diaphoresis (3%), hair loss (2%), diarrhea (2%), ageusia (2%), and nausea (2%) (Table 2).

**Table 2:** Severity and prevalence of symptoms at D28+ survey (N = 411)

Symptom	Mild	Moderate	Severe	Overall prevalence*
Cough	20.4%	7.8%	1.2%	29.4%
Headache	17.3%	9.0%	1.7%	28.0%
Myalgia	15.3%	8.8%	1.9%	26.0%
Anosmia	20.2%	NA	3.4%	23.6%
Dyspnea	14.6%	7.8%	0.7%	23.1%
Post-exertion polypnea	12.7%	7.4%	1.0%	21.1%
Ageusia	18.0%	NA	2.2%	20.2%
Cognitive disturbances	11.2%	6.6%	1.7%	19.5%
Chest pain/tightness	12.4%	4.4%	0.7%	17.5%
Arthralgia	7.1%	6.1%	2.0%	15.1%
Hair loss	6.6%	5.1%	2.4%	14.1%
Palpitations	6.3%	5.1%	0.7%	12.2%
Nausea	6.1%	3.2%	2.2%	11.4%
Diaphoresis	4.6%	3.4%	2.7%	10.7%
Rhinorrhea	8.0%	2.2%	0.2%	10.5%
Pharyngitis	7.5%	1.5%	0.5%	9.5%
Ear pain	6.6%	2.2%	0.7%	9.5%
Diarrhea	3.7%	2.2%	2.4%	8.3%
Rash	3.9%	2.0%	0.5%	6.3%
Chills/shivering	4.6%	1.2%	0.2%	6.1%
Need for oxygen	2.4%	2.4%	1.0%	5.9%
Loss of hearing	4.0%	NA	0.0%	4.0%
Fever	2.4%	0.2%	0.5%	3.2%
Vomiting	1.9%	0.0%	0.5%	2.4%
Thromboembolism	0.5%	1.0%	0.2%	1.7%
CKD	0.7%	0.2%	0.2%	1.2%

For diarrhea and vomiting, 1–2 was classified as mild, 3–4 as moderate, and 5 or more as severe. For loss of hearing, smell, and taste, “same as usual” was considered none, “less than usual” was considered mild, and “no sense” was considered severe

*CKD* chronic kidney disease, *NA* not applicable

*Overall prevalence represents the sum of mild, moderate, and severe categories

### Longitudinal findings

**Fig. 2:**
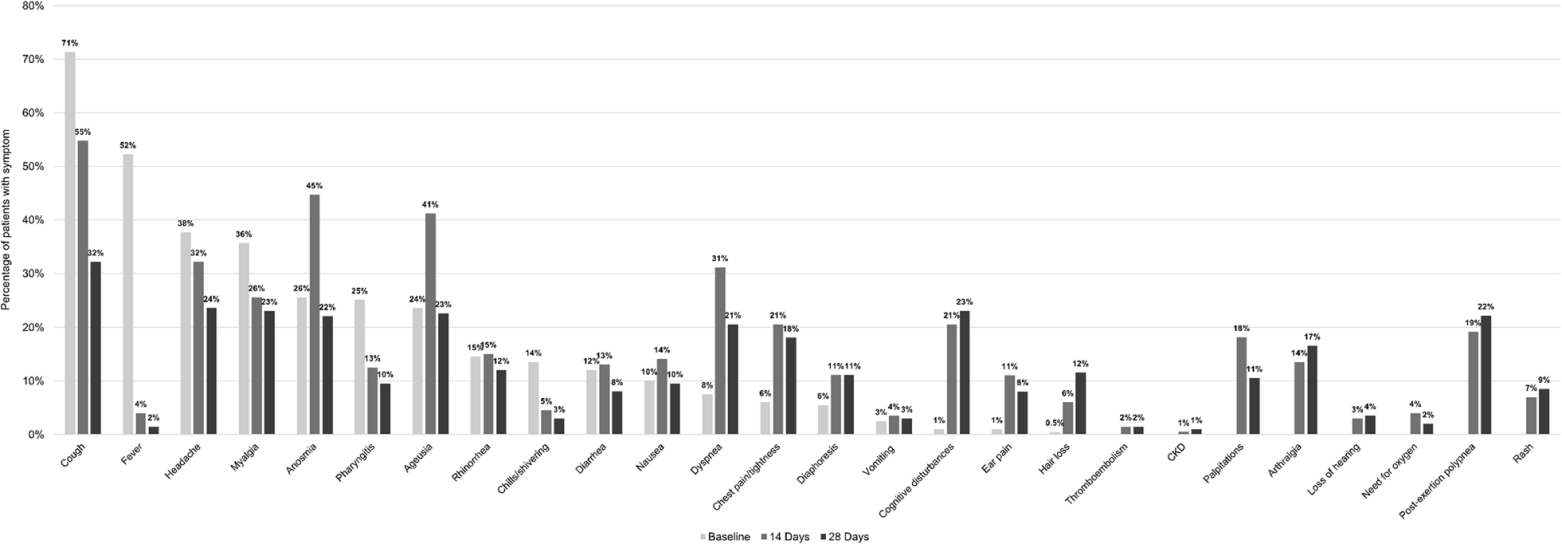
Symptom prevalence at baseline vs. D14 and D28+ surveys (N = 199). Among patients who completed the Baseline, D14, and D28+ surveys (N = 199). Follow-up times for the surveys varied. At baseline, no patients reported having the following symptoms: thromboembolism, CKD, palpitations, arthralgia, loss of hearing, need for oxygen, post-exertion polypnea, and rash. *CKD* chronic kidney disease

**Fig. 3:**
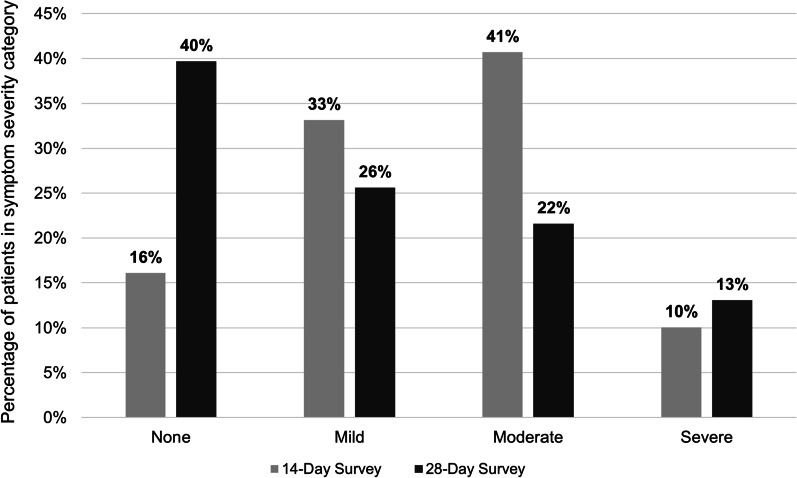
Symptom severity at D14 vs. D28+ surveys (N = 199). Among patients who completed the Baseline, D14, and D28+ Surveys. Follow-up times for the surveys varied. “None” defined as no mild, moderate, or severe symptoms; “Mild” defined as ≥ 1 mild symptom (and no moderate or severe symptoms); “Moderate” defined as ≥ 1 moderate symptom (and no severe symptoms); “Severe” defined as ≥ 1 severe symptom. The following symptoms were excluded from calculations because of how their severity levels were classified: vomiting, diarrhea, loss of hearing, anosmia, and ageusia

Among patients who completed all three surveys (N = 199), at D14 the most prevalent symptoms were cough (55%), anosmia (45%), ageusia (41%), headache (32%), and dyspnea (31%). At D28+ , the most prevalent symptoms were cough (32%), headache (24%), cognitive disturbances (23%), myalgia (23%), ageusia (22%), and post-exertion polypnea (22%) (Fig. 2). The largest changes in prevalence over time (from Baseline to D28+) occurred in the following symptoms: fever (− 50%), cough (− 39%), cognitive disturbances (+ 22%), and post-exertion polypnea (+ 22%). The percentage of patients with no symptoms increased from D14 (16%) to D28+ (40%) (Fig. 3). The percentages of patients with mild and moderate symptoms each decreased from D14 (33% and 41%) to D28+ (26% and 21%). At D14, 10% of patients had severe symptoms compared to 13% at D28+.

Additional longitudinal outcomes included the change in PHQ-2 Total and PROMIS-29 Domain Scores over time. Mean PHQ-2 Total and PROMIS-29 Domain scores at D28+ are shown in Table 3.

**Table 3:** PHQ-2 and PROMIS-29 domain scores at D28 + survey (N = 411)

Measure*	Mean (SD)
PHQ-2 total score	0.5 (1.1)
PROMIS-29 domain scores^**^	
Physical function	18.1 (3.2)
Fatigue	7.9 (4.2)
Pain interference	6.2 (3.7)
Anxiety	4.4 (2.5)
Sleep disturbance	9.3 (4.0)
Depression	5.1 (2.6)
Social roles	13.6 (2.8)

*PHQ-2* Patient Health Questionnaire-2, *PROMIS-29* Patient-Reported Outcomes Measurement Information System-29

*PHQ-2 Total Score range is 0 to 6; PROMIS-29 Domain Score ranges are 0 to 20

**Higher scores indicate better functioning on Physical Function and Social Roles Domains. Higher scores represent worse symptomology on Fatigue, Pain Interference, Anxiety, Sleep Disturbance, and Depression Domains

**Fig. 4:**
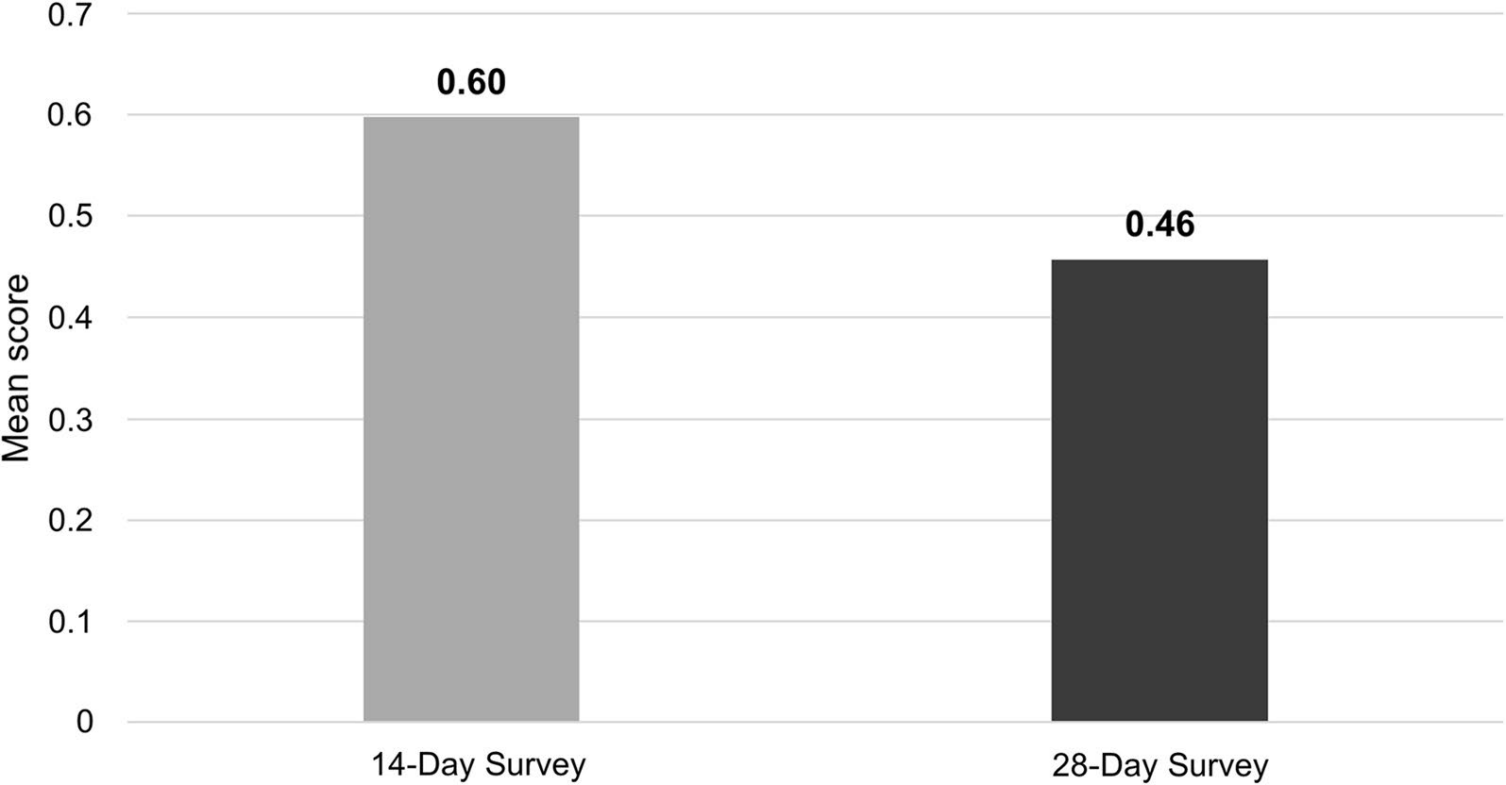
PHQ-2 total scores at D14 vs. D28+ surveys (N = 199). PHQ-2 Total Score range is 0 to 6. PHQ-2, Patient Health Questionnaire-2

**Fig. 5:**
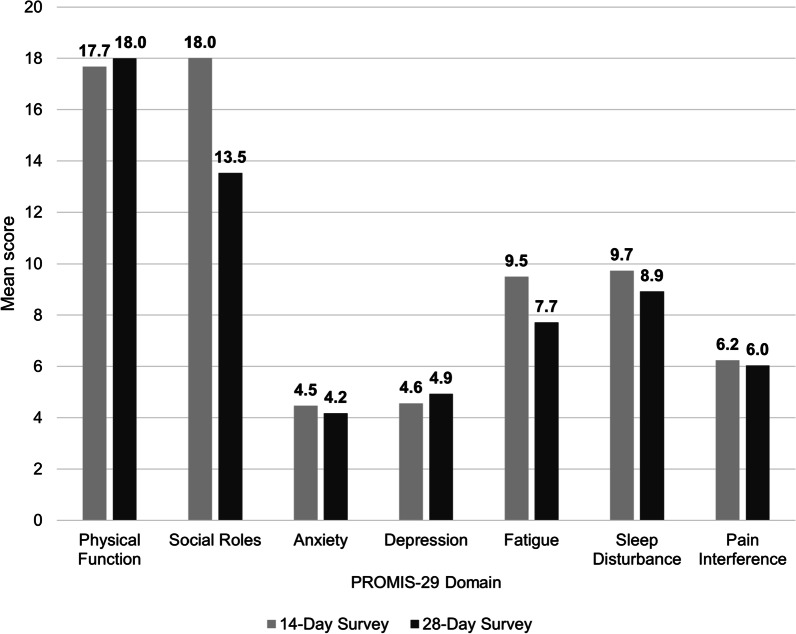
PROMIS-29 domain scores at D14 vs D28+ surveys (N = 199). PROMIS-29 Domain Score ranges are 0 to 20. Higher scores indicate better functioning on Physical Function and Social Roles Domains. Higher scores represent worse symptomology on Anxiety, Depression, Fatigue, Sleep Disturbance, and Pain Interference Domains. PROMIS-29, Patient-Reported Outcomes Measurement Information System-29

Mean PHQ-2 Total Scores decreased from D14 to D28+ (Fig. 4). For the two PROMIS-29 Domains where higher scores indicate better functioning, mean scores increased from D14 to D28+ for Physical Function but decreased for Social Roles (Fig. 5). For four out of five of the PROMIS-29 Domains where higher scores represent worse symptomology (Anxiety, Fatigue, Sleep Disturbance, and Pain Interference), mean scores decreased from D14 to D28+. Mean scores for the PROMIS-29 Depression Domain increased from D14 to D28+.

### Time to mAb infusion

Results of the tests of association between TTI and outcomes of interest at D28+ showed that TTI was not statistically significantly associated with any outcome (symptom count category, symptom severity category, PCC status [by any of 6 PCC definitions], PHQ-2 Total Score, or any PROMIS-29 Domain Score (Table 4).

**Table 4:** Tests of association between TTI and outcomes at D28+ survey (N = 409)

Predictor variable	Outcome variable	P-value
*Pearson’s Chi-squared tests*		
Time to infusion	Symptom count category	0.6569
Time to infusion	Symptom severity category	0.3205
Time to infusion	PCC status (Definition 1)	0.8362^a^
Time to infusion	PCC status (Definition 2)	0.7404^a^
Time to infusion	PCC status (Definition 3)	0.8200^a^
Time to infusion	PCC status (Definition 4)	0.3765^a^
Time to infusion	PCC status (Definition 5)	0.1582^a^
Time to infusion	PCC status (Definition 6)	0.7731^a^
*Independent 2-sample t-tests*		
Time to infusion	PHQ-2 total score	0.2991
Time to infusion	PROMIS-29 physical function	0.4223^b^
Time to infusion	PROMIS-29 fatigue	0.1553^b^
Time to infusion	PROMIS-29 pain interference	0.0279^b^
Time to infusion	PROMIS-29 anxiety	0.8561
Time to infusion	PROMIS-29 sleep disturbance	0.0781^b^
Time to infusion	PROMIS-29 depression	0.9668
Time to infusion	PROMIS-29 social roles	0.3350^b^

Pearson’s Chi-squared tests performed for categorical outcomes; 2-sample independent t-tests performed for continuous outcomes. Significance level (alpha) of 0.05 for Pearson’s Chi-squared tests. Bonferroni correction used for 8 independent 2-sample t-tests—significance level (alpha) of 0.00625

Symptom count categories defined by total number of symptoms at Day 28 Survey (0, 1–2, 3–4, or 5–20)

Symptom severity categories defined by total number of moderate/severe symptoms at Day 28 Survey (0, 1, 2, ≥ 3)

Time to infusion defined as the time between symptom onset and mAb infusion. Treated as a binary variable with groups: 1–6 days (n = 203) vs 7–10 days (n = 206)

Original N = 411; tests exclude 2 patients with outlier times to infusion

*PHQ-2* Patient Health Questionnaire-2, *PROMIS-29* Patient-Reported Outcomes Measurement Information System-29, *TTI* time to mAb infusion

^a^P-value for Pearson's Chi-squared test with Yates' continuity correction

^b^P-value from Welch’s t-test due to unequal variances. Otherwise, Student’s t-test (assumes equal variances) performed

### Prevalence of PCC

Based on Definition 1 (≥ 1 symptom at D28+), the prevalence of PCC in the study population was 69%; based on Definition 2 (≥ 3 symptoms), the prevalence was 44%; based on Definition 3 (≥ 5 symptoms), the prevalence was 30% (Table 5). Based on Definition 4 (≥ 1 moderate or severe symptom), the prevalence of PCC was 47%; based on Definition 5 (≥ 2 moderate or severe symptoms), the prevalence was 36%; based on Definition 6 (≥ 3 moderate or severe symptoms), the prevalence was 23%.

**Table 5:** Prevalence of PCC at D28+ survey (N = 403)

PCC definition	Prevalence of PCC, n (%)
*Definitions based on number of symptoms*	
PCC Definition 1 (≥ 1 symptom)	277 (68.7%)
PCC Definition 2 (≥ 3 symptoms)	178 (44.2%)
PCC Definition 3 (≥ 5 symptoms)	121 (30.0%)
*Definitions based on severity of symptoms*	
PCC Definition 4 (≥ 1 moderate or severe symptom)	188 (46.5%)
PCC Definition 5 (≥ 2 moderate or severe symptoms)	145 (35.9%)
PCC Definition 6 (≥ 3 moderate or severe symptoms)	93 (23.0%)

Based on 28 days or more post-symptom onset; follow-up times for D28+ survey varied

Original N = 411; calculations exclude 8 patients with time from symptom onset to Day 28 survey < 28 days

*PCC* post-COVID conditions

### Time to mAb infusion as a predictor of PCC

The Poisson regression produced crude and adjusted prevalence ratios of PCC based on TTI. In the crude (unadjusted) model, patients with TTI of 7 to 10 days had 1.02 times the risk of developing PCC compared to patients with TTI of 1 to 6 days (95% confidence interval [CI]: 0.90 to 1.16; P = 0.75) (Table 6). In Model 1, which adjusted for demographic characteristics only, the prevalence ratio was 1.02 (95% CI: 0.89 to 1.16; P = 0.79. In Model 2, which adjusted for both demographic and clinical characteristics, the prevalence ratio was 1.01 (95% CI: 0.89 to 1.16; P = 0.84). Time to mAb infusion was not statistically significantly associated with PCC status at D28 + in any of the models.

**Table 6:** Prevalence ratio of PCC for TTI of 7–10 days versus 1–6 days (N = 409)

Model	PR	95% CI	P-value
Crude (unadjusted)	1.02	0.90 to 1.16	0.75
Model 1: crude + demographic characteristics*	1.02	0.89 to 1.16	0.79
Model 2: model 1 + clinical characteristics**	1.01	0.89 to 1.16	0.84

Modified Poisson regressions with robust standard errors performed. Results are for time to infusion of 7–10 days (n = 206) compared to the reference group, time to infusion of 1–6 days (n = 203). PCC was defined by PCC Definition 1 (≥ 1 symptom at D28 + Survey). Original N = 411; analysis excludes 2 patients with outlier times to infusion

*CI* confidence interval, *PCC* post-COVID conditions, *PR* prevalence ratio, *TTI* time to mAb infusion

*Model 1 was adjusted for demographic characteristics (age, sex assigned at birth, race, and ethnicity) only

**Model 2 was adjusted for demographic characteristics (age, sex assigned at birth, race, and ethnicity) plus clinical characteristics (vaccination status pre-infusion, mAb infusion type, and number of symptoms at Baseline Survey)

## Discussion

Cross-sectional results among the entire study population (N = 411) uncovered an initially high symptom burden—at baseline, 70% of patients had cough, 50% had fever, 44% had headache, 40% had fatigue, and 38% had myalgia. The prevalence of many symptoms markedly decreased by the time of D28+, when only 29% of patients had cough, 3% had fever, 28% had headache, and 26% had myalgia. With respect to symptom severity at D28+, mild symptoms were most common (≤ 20% of patients), moderate symptoms were less common (≤ 9%), and severe symptoms were rare (≤ 3%).

Longitudinal results among patients who completed all three surveys (N = 199) showed that from Baseline to D28+, 10 symptoms decreased in prevalence (cough, fever, headache, myalgia, anosmia, pharyngitis, ageusia, rhinorrhea, chills/shivering, and diarrhea), 2 remained the same (nausea and vomiting), and 14 increased (dyspnea, chest pain/tightness, diaphoresis, cognitive disturbances, ear pain, hair loss, thromboembolism, CKD, palpitations, arthralgia, loss of hearing, need for oxygen, post-exertion polypnea, and rash). The symptoms with the largest decreases in prevalence from Baseline to D28+ were cough and fever; cognitive disturbances and post-exertion polypnea had the largest increases in prevalence over time. Overall, the severity of symptoms decreased over time—at D14 most patients either had mild (33%) or moderate (41%) symptoms, while at D28+ a large percentage of patients had no symptoms (40%). With respect to change in patient-reported physical and mental health measures from D14 to D28+, PHQ-2 Total scores decreased over time and scores on most PROMIS-29 Domains (all except Social Roles and Depression) improved over time.

The prevalence of PCC was notably high—69% when PCC was defined as ≥ 1 symptom at D28+. Even when defined by extreme terms (i.e., ≥ 5 symptoms or ≥ 3 moderate or severe symptoms at D28+), the prevalence of PCC was still high (30% and 23%, respectively). Time to mAb infusion was not significantly associated with symptom count category, symptom severity category, PCC status, PHQ-2 Total score, or any PROMIS-29 Domain score. Time to infusion was not associated with PCC status at D28+ in any of the models from the Poisson regression analysis, crude or adjusted. Thus, our findings did not support our hypothesis that a shorter TTI would reduce the risk of PCC.

While evidence supports the effectiveness of mAbs at reducing hospitalization and mortality rates in real-world settings, most studies have focused on clinical outcomes rather than patient-centric outcomes such as symptom burden [5–8, 10, 11, 32–34]. Findings are mixed regarding symptom burden over time, with several studies reporting a reduction in symptom duration or time to symptom resolution with mAbs but another study reporting that mAbs did not shorten symptom duration [35–37]. Patients receiving mAbs may experience a slight decrease in symptom severity compared to those receiving placebo [9]. A recent Cochrane review identified a lack of evidence on clinical progression, improvement of symptoms, and development of severe symptoms among non-hospitalized patients treated with mAbs [38]. In the present study, the high prevalence of symptoms at follow-up (i.e., the fact that seven symptoms had a prevalence ≥ 20% at D28+) suggests that patients with COVID-19 receiving mAbs experience a substantial and ongoing symptom burden. The finding that very few patients had severe symptoms at follow-up (i.e., no severe symptom had a prevalence > 3% at D28+) suggests that patients’ symptoms did improve over time. Since this descriptive study lacked a control group, these results shed light on the evolution of symptoms among high-risk patients receiving mAbs but cannot provide insight into the impact of mAbs. Future studies that directly compare mAbs to placebo or an untreated control group will be important to establish whether mAbs improve symptom resolution over time. The authors are not aware of any existing studies evaluating patient-reported outcomes (e.g., PROMIS-29, SF-36, or PHQ-2) among patients with COVID-19 treated with mAbs. While the present study explores these outcomes, research into the actual effect of mAbs on patients’ mental health and quality of life is warranted.

While mAbs are thought to be more effective at reducing symptom duration during the initial phase of illness while the SARS-CoV2 virus undergoes replication in the body, a higher level of effectiveness when administered sooner after symptom onset has not been definitively established [37, 39]. Evidence from recent studies supports the existence of this association between faster TTI and improved outcomes [10, 11, 33]. Administration of mAbs within 6 days of symptom onset demonstrated higher effectiveness at preventing hospitalizations and ED visits among high-risk outpatients [11]. Hospitalization rates were positively correlated with increasing TTI [33]. Among solid organ transplant recipients, TTI differed significantly between hospitalized and non-hospitalized patients, suggesting that earlier mAb infusion may reduce hospitalizations [10]. In contrast, the present study found that TTI was not associated with patient outcomes at follow-up. Future studies should compare hospitalizations, mortality, and symptom resolution between patients receiving mAbs earlier versus later following symptom onset—further research is essential to determine the ideal timing of mAbs to improve clinical outcomes [33].

Efforts to better understand the burden of PCC among COVID-19 patients are ongoing, and the prevalence of PCC specifically among patients treated with mAbs is unexplored. A recent CDC study estimated that approximately 1 in 5 COVID-19 survivors aged 18 to 64 and 1 in 4 survivors aged ≥ 65 had PCC [40]. This mirrors the finding from a previous CDC study that 33% of patients who recovered from COVID-19 had PCC [19]. Two different systematic reviews and meta-analyses estimated a prevalence of ≤ 80% [14, 41]. At 30 days from symptom onset, studies have placed the prevalence of PCC between 53 and 63% [16, 42]. The present study found a PCC prevalence of 69% at D28+ , mirroring the findings of studies conducted in patients not treated with mAbs and supporting a growing body of evidence that the burden of PCC is much greater than initially thought. Our finding of such a high prevalence of PCC is particularly interesting given the follow-up times to D28+ (mean 72 days; range 21 to 186). The question of whether a more rapid TTI reduces a patient’s risk of developing PCC is unexplored in research [37]. The present study did not find evidence supporting the notion that time to mAb infusion predicts risk of developing PCC. However, given the body of evidence that shorter TTI results in improved clinical outcomes (e.g., reduced hospitalizations), it is possible that a shorter TTI may be associated with faster symptom resolution and, consequently, a lower prevalence of PCC [10, 11]. Future studies should directly examine PCC prevalence based on differential TTI.

This study is not without limitations. The observational design did not allow for the inclusion of a control group of patients with COVID-19 who did not receive mAbs, preventing us from drawing any conclusions on the impact of mAbs. Although the study was conducted in an FQHC setting that made mAb infusions accessible to a predominately low-income and Latino/Hispanic population, data on certain variables (e.g., vaccination status) were limited. We were unable to differentiate between SARS-CoV-2 variants, which are differentially susceptible to neutralization by mAbs, though we can assume that our participants had variants other than Omicron (the dates of mAb infusions ranged from 12/31/2020 to 10/5/2021, before the first Omicron infection in San Diego County was identified) [39, 43]. Our mAb clinic did change use of mAbs to match local variant prevalence and susceptibility patterns according to NIH guidelines. There was wide variation in follow-up times from symptom onset to D28+ (mean 72 days; range 21 to 186); while the mean of 72 days is close to WHO’s definition of PCC as 90 days, a longer follow-up would have been preferable to assess post-COVID symptoms. Due to the survey design, all data in this study were self-reported, leading to potential information bias [44]. Some form of selection bias may have occurred, including non-response bias (patients with more severe disease may have been less likely to agree to participate in the study) and differential loss to follow-up (participants with more severe disease or who died would be lost to follow-up at higher rates) [45]. Furthermore, patients with mild COVID-19 may have been less likely to present for care and mAb infusion. While we were able to use well-validated measures of patient outcomes (PROMIS-29 and PHQ-2) and define PCC by six definitions of varying stringencies to provide deeper insight into the burden of ongoing COVID-19 symptoms, we were not able to incorporate data on hospitalizations or deaths. Additionally, we were unable to adjust for comorbidities that increase the risk for severe illness with COVID-19 (e.g., cancer, CVD, chronic lung diseases, diabetes, and human immunodeficiency virus [HIV], among others) in our regression models [46]. Since our patient population included those at high risk for serious outcomes with COVID-19, it is reasonable to assume that many had these comorbidities. This renders our results regarding symptoms and PCC generalizable to populations with comorbidities, rather than the general population.

In summary, this unique study focused on patient-centric outcomes including symptom burden over time and PCC. It fills a gap in existing research by identifying a high prevalence of PCC among patients who have received mAb treatment for COVID-19. Further, findings do not support the hypothesis that time to infusion is associated with PCC. Given that unanswered questions about both PCC and time to mAb infusion have important implications for clinical practice and population health management of both COVID-19 and PCC, it is vital that further research pursues deeper understanding of these timely topics.

## Data Availability

The datasets used and/or analyzed during the current study are available from the corresponding author on reasonable request.

## Abbreviations

COVID-19: Coronavirus disease 2019
US: United States
FDA: Food and Drug Administration
EUAs: Emergency use authorizations
mAbs: Monoclonal antibodies
SARS-CoV-2: Severe acute respiratory syndrome coronavirus 2
TTI: Time to infusion
BMI: Body mass index
CKD: Chronic kidney disease
CVD: Cardiovascular disease
CDC: Centers for Disease Control and Prevention
PCC: Post-COVID conditions
WHO: World Health Organization
FHCSD: Family Health Centers of San Diego
FQHC: Federally qualified health center
MASS: Monoclonal Antibody Screening Score
NIH: National Institutes of Health
LRRI: Laura Rodriguez Research Institute
D14: COVID mAb Follow-up Day 14 Survey
D28+: COVID mAb Follow-up Day 28+ Survey
PHQ-2: Patient Health Questionnaire-2
PROMIS-29: Patient-Reported Outcomes Measurement Information System-29
HIV: Human immunodeficiency virus
